# A gene-edited mouse model of limb-girdle muscular dystrophy 2C for testing exon skipping

**DOI:** 10.1242/dmm.040832

**Published:** 2019-11-04

**Authors:** Alexis R. Demonbreun, Eugene J. Wyatt, Katherine S. Fallon, Claire C. Oosterbaan, Patrick G. Page, Michele Hadhazy, Mattia Quattrocelli, David Y. Barefield, Elizabeth M. McNally

**Affiliations:** 1 Center for Genetic Medicine, Northwestern University, Chicago, IL 60611, USA; 2 Department of Pharmacology, Northwestern University, Chicago, IL 60611, USA

**Keywords:** LGMD 2C, Antisense oligonucleotide, Sarcoglycan, Dystrophin, Gene correction, Mouse

## Abstract

Limb-girdle muscular dystrophy type 2C is caused by autosomal recessive mutations in the γ-sarcoglycan (*SGCG*) gene. The most common *SGCG* mutation is a single nucleotide deletion from a stretch of five thymine residues in *SGCG* exon 6 (521ΔT). This founder mutation disrupts the transcript reading frame, abolishing protein expression. An antisense oligonucleotide exon-skipping method to reframe the human 521ΔT transcript requires skipping four exons to generate a functional, internally truncated protein. *In vivo* evaluation of this multi-exon skipping, antisense-mediated therapy requires a genetically appropriate mouse model. The human and mouse γ-sarcoglycan genes are highly homologous in sequence and gene structure, including the exon 6 region harboring the founder mutation. Herein, we describe a new mouse model of this form of limb-girdle muscular dystrophy generated using CRISPR/Cas9-mediated gene editing to introduce a single thymine deletion in murine exon 6, recreating the 521ΔT point mutation in *Sgcg*. These mice express the 521ΔT transcript, lack γ-sarcoglycan protein and exhibit a severe dystrophic phenotype. Phenotypic characterization demonstrated reduced muscle mass, increased sarcolemmal leak and fragility, and decreased muscle function, consistent with the human pathological findings. Furthermore, we showed that intramuscular administration of a murine-specific multiple exon-directed antisense oligonucleotide cocktail effectively corrected the 521ΔT reading frame. These data demonstrate a molecularly and pathologically suitable model for *in vivo* testing of a multi-exon skipping strategy to advance preclinical development of this genetic correction approach.

## Abstract (precis)

**Summary:** The most common *SGCG* mutation that causes LGMD 2C has been modeled in mice using gene editing, providing a platform for preclinical evaluation of multi-exon antisense therapy.

## INTRODUCTION

Limb-girdle muscular dystrophy type 2C (LGMD 2C) is a rare genetic disorder caused by autosomal recessive mutations in the γ-sarcoglycan (*SGCG*) gene (McNally et al., 1996b; Noguchi et al., 1995). γ-Sarcoglycan is a dystrophin-associated protein, and loss of function mutations in the *SGCG* gene produces a clinical picture similar to what is seen in Duchenne muscular dystrophy (DMD) (Ben Jelloun-Dellagi et al., 1990; Matsumura et al., 1992). There is currently no effective treatment for this form of LGMD, and clinical care focuses on alleviating symptoms by providing respiratory and cardiac support (Straub and Bushby, 2008).

The *SGCG* gene itself is comprised of eight exons and encodes γ-sarcoglycan, a type II transmembrane protein, expressed highly in skeletal and cardiac muscle. γ-Sarcoglycan is an essential component of a large membrane-linked dystrophin-associated protein complex (Cohn and Campbell, 2000; Ervasti and Campbell, 1993). This complex is required for muscle membrane stability and function, and its disruption results in muscle degeneration and wasting (Durbeej and Campbell, 2002; Rahimov and Kunkel, 2013). Mutations that disrupt the dystrophin gene cause DMD, the most common form of muscular dystrophy (Hoffman et al., 1987). Recently, the United States Food and Drug Administration (FDA) approved a first-in-class exon-skipping gene therapy to treat the most common dystrophin mutations, directly targeting the underlying genetic cause of disease (Aartsma-Rus and Krieg, 2017).

Exon skipping relies on chemically modified antisense oligonucleotides (AONs) to modulate gene expression, including correction of an aberrant reading frame which abrogates protein expression (Chan et al., 2006). Chemically modified AONs can modulate pre-mRNA splicing, bypassing mutations and generating an internally truncated protein that is able to partially rescue the loss of the full-length protein (Kole et al., 2012). In DMD, internally deleted forms of dystrophin are known to be functional and result in a milder form of disease known as Becker muscular dystrophy (BMD) (Koenig et al., 1989; Monaco et al., 1988). The goal of exon skipping to treat DMD is to induce retention of out-of-frame exons and to create an intact reading frame. The development of antisense-mediated splice modulating therapy is expanding beyond DMD to other disorders including Pompe disease, cystic fibrosis, cardiomyopathies and laminopathies (Clayton et al., 2014; Gedicke-Hornung et al., 2013; Gramlich et al., 2015; Igreja et al., 2016).

Recently, we described an exon skipping strategy to treat LGMD 2C (Gao et al., 2015; Wyatt et al., 2018). The most common mutation resulting in LGMD 2C is a single thymine deletion in *SGCG* exon 6, designated 521ΔT (McNally et al., 1996a; Noguchi et al., 1995). This founder mutation disrupts the reading frame, ablating γ-sarcoglycan protein expression. Reading frame correction requires skipping of exons 4, 5, 6 and 7 to create an internally truncated protein termed Mini-Gamma. Mini-Gamma is encoded by exons 2, 3 and 8 (Gao et al., 2015; Wyatt et al., 2018). Transgenic overexpression of Mini-Gamma protein in *Drosophila* and mice lacking γ-sarcoglycan rescued the dystrophic phenotype (Gao et al., 2015). Furthermore, a multi-exon skipping cocktail was able to correct *SGCG* mutations in cell lines derived from LGMD 2C patients (Wyatt et al., 2018).

Although these previous studies demonstrate aspects of the feasibility of multi-exon skipping, the lack of preclinical *in vivo* testing has been a limitation. A previously generated γ-sarcoglycan-null mouse model resembles findings seen in human patients with sarcolemmal fragility, muscle degeneration and impaired muscle function (Hack et al., 1998). However, the mouse models in which the γ-sarcoglycan gene was disrupted lack the exon containing the initiator methionine, and are not amenable to exon skipping (Hack et al., 1998; Sasaoka et al., 2003). The human and mouse γ-sarcoglycan genes share >80% homology, including the stretch of five thymine residues in exon 6. We used CRISPR/Cas9 technology with homology-directed repair to introduce the specific 521ΔT mutation in mice. These mice express the mutant 521ΔT transcript and lack γ-sarcoglycan expression. Furthermore, they exhibit a severe dystrophic phenotype, which includes muscle degeneration, increased membrane leak and fibrosis, and loss of muscle function. This is a suitable model for preclinical evaluation of multi-exon skipping therapy to treat the majority of people with LGMD 2C.

## RESULTS

### Generation of 521ΔT mice

**Fig. 1.**
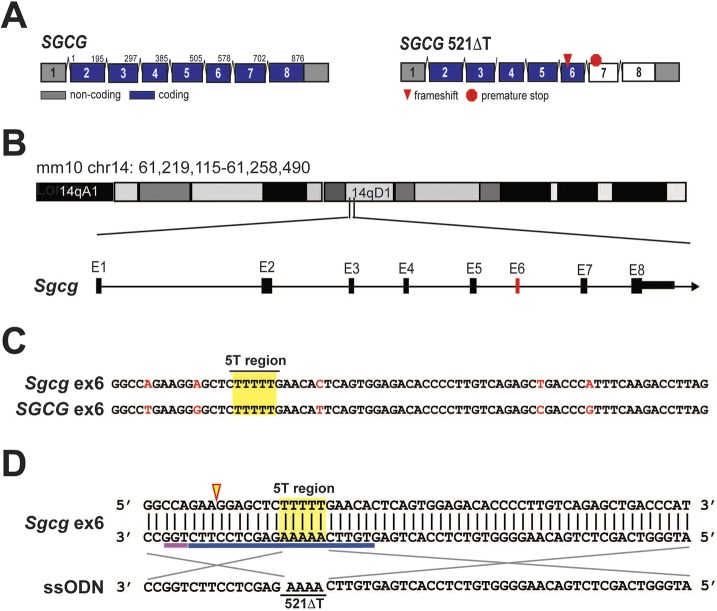
**Gene editing to engineer the 521ΔT *Sgcg* point mutation in mice.** (A) Schematic of the human *SGCG* gene on the left and the 521ΔT mutation on the right. The 521ΔT mutation is the most common LGMD 2C mutation. The single thymine deletion causes a frameshift, the expression of a premature stop codon, and loss of γ-sarcoglycan protein expression. (B) Schematic of the genetic locus and composition of the murine *Sgcg* gene. Both the human and the mouse genes include eight exons, with exons 2-8 encoding protein. Each coding region is 876 bp with each individual exon having the same number of coding base pairs. Exon 6 is highlighted in red. (C) Both human and murine exon 6 are 73 bp in length, and each has a stretch of five thymines in the same location (highlighted in yellow). Individual base pair mismatches are shown in red. (D) Schematic depicting the CRISPR design strategy to introduce the 521ΔT mutation into the mouse genome. A 20 nt gRNA sequence (blue line) was used to direct Cas9 nuclease to the specific locus, defined by the presence of a -NGG protospacer adjacent motif (PAM, pink line). The nuclease created a double- strand break 3 bp upstream of the PAM (arrowhead). The addition of a 180 nt repair template promoted HDR and contained the single base pair deletion (gray lines depict homology arms).

The most common γ-sarcoglycan mutation in humans, 521ΔT, disrupts the *SGCG* reading frame in exon 6, causing loss of protein expression (Fig. 1A) (McNally et al., 1996a; Noguchi et al., 1995). The mouse *Sgcg* gene is located on chromosome 14 (Fig. 1B), and both the human and mouse γ-sarcoglycan genes share a similar gene structure, with eight exons, sharing more than 80% homology at the genomic and protein levels. This homology extends to the 521ΔT region of exon 6 (Fig. 1C). The advent of CRISPR/Cas technology has significantly impacted the ease of generating mouse models of human disease (Wang et al., 2013). A single-guide RNA (gRNA) can direct double-strand breaks in the genome. Precise genome edits can be achieved through homology directed repair (HDR) when accompanied by a repair template (Cong et al., 2013). We used CRISPR/Cas9 technology to engineer a new mouse model of LGMD 2C, caused by a single thymine deletion in *Sgcg* exon 6, termed 521ΔT. The specific design is shown in Fig. 1D.

CRISPR reagents were microinjected into zygotes derived from C57BL/6J mice, and introduced into pseudo-pregnant females. Of 13 potential founders, a single mouse harbored a heterozygous 521ΔT mutation in the *Sgcg* locus, with the other allele remaining unedited [wild type (WT)]. This F0 mouse was crossed with a WT C57BL/6J mouse (Fig. S1). Heterozygous offspring were then backcrossed for five generations onto the DBA/2J background. The DBA/2J genetic background strain has previously been reported to modify the dystrophic phenotype, resulting in a more severe disease progression owing to polymorphisms in modifier genes, including an in-frame deletion in the latent TGFβ-binding protein 4 (*Ltbp4*) gene, which is not present in the C57 background (Fig. S1) (Coley et al., 2016; Heydemann et al., 2009; Swaggart et al., 2014). The 521ΔT mice from the F5 generation were genotyped for the deleterious *Ltbp4* allele and were homozygous for the severe *Ltbp4* allele (Fig. S1). Homozygous 521ΔT mice were born in the expected ratio (at 28% versus the expected 25%) (Fig. S1).

### Sgcg expression in 521ΔT muscle

**Fig. 2.**
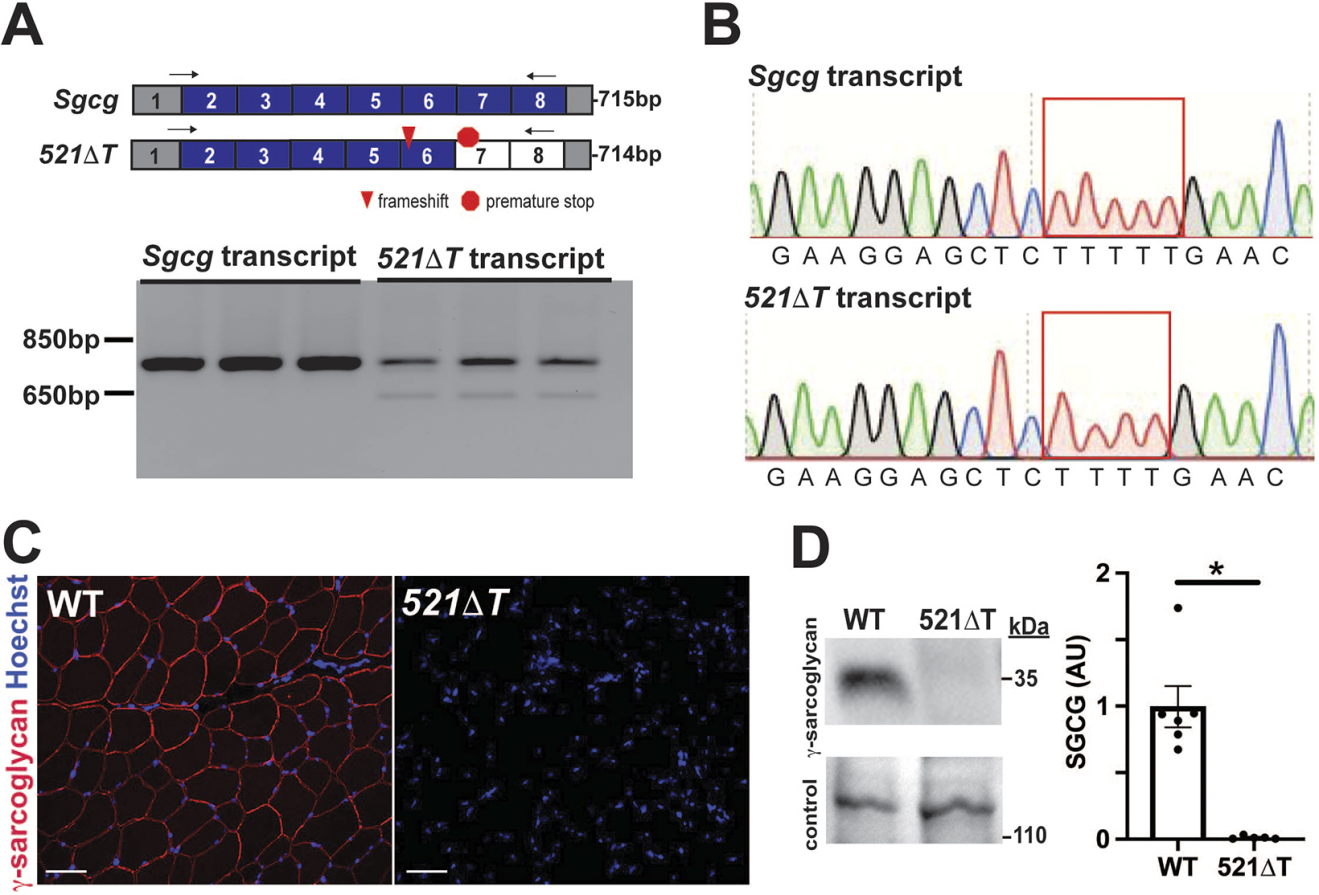
**Reduction of *Sgcg* transcript and loss of γ-sarcoglycan protein expression in *Sgcg* 521ΔT muscle.** (A) RT-PCR illustrating the presence of *Sgcg* (715 bp) and *Sgcg* 521ΔT (714 bp) transcripts. (B) Sequence chromatograms of the 5T region in the *Sgcg* and *Sgcg* 521ΔT transcripts illustrating the deletion of one thymine in exon 6 (red box) of the *Sgcg* 521ΔT transcript. (C) γ-Sarcoglycan protein (red) was readily detected in WT muscle, but not in 521ΔT muscle. Hoechst (blue) marked nuclei. (D) Immunoblot analysis revealed the presence of γ-sarcoglycan protein in WT muscle lysates, whereas γ-sarcoglycan was not detected in 521ΔT muscle lysates. Data are mean±s.e.m. *P<0.05 (*t*-test). Scale bars: 50 µm.

To validate that the deletion of the single thymine in exon 6 resulted in loss of γ-sarcoglycan, we evaluated gene and protein expression. RT-PCR analysis documented a reduction in *Sgcg* transcript in 521ΔT muscle, compared with WT. An additional smaller transcript was seen at 590 bp, and this shorter transcript represents endogenous skipping of exon 7 in 521ΔT muscle (Fig. S2), similar to previous reports in human cell lines (Wyatt et al., 2018). Sequence analysis of RT-PCR products showed deletion of a single thymine in the 5 T region of exon 6 in the *521*Δ*T* transcript (Fig. 2A,B). The deletion results in a premature stop codon and this resulted in absence of γ-sarcoglycan protein (Fig. 2C,D). We examined expression of γ-sarcoglycan in muscle using immunofluorescence microscopy and confirmed loss of γ-sarcoglycan from its normal position at the plasma membrane (Fig. 2C). Loss of γ-sarcoglycan was also observed by immunoblot analysis (Fig. 2D). These data demonstrate that a single thymine deletion in exon 6 results in loss of γ-sarcoglycan protein, identical to that seen in human LGMD 2C patients.

### Reduced mass and extensive muscle pathology in 521ΔT mice

**Fig. 3.**
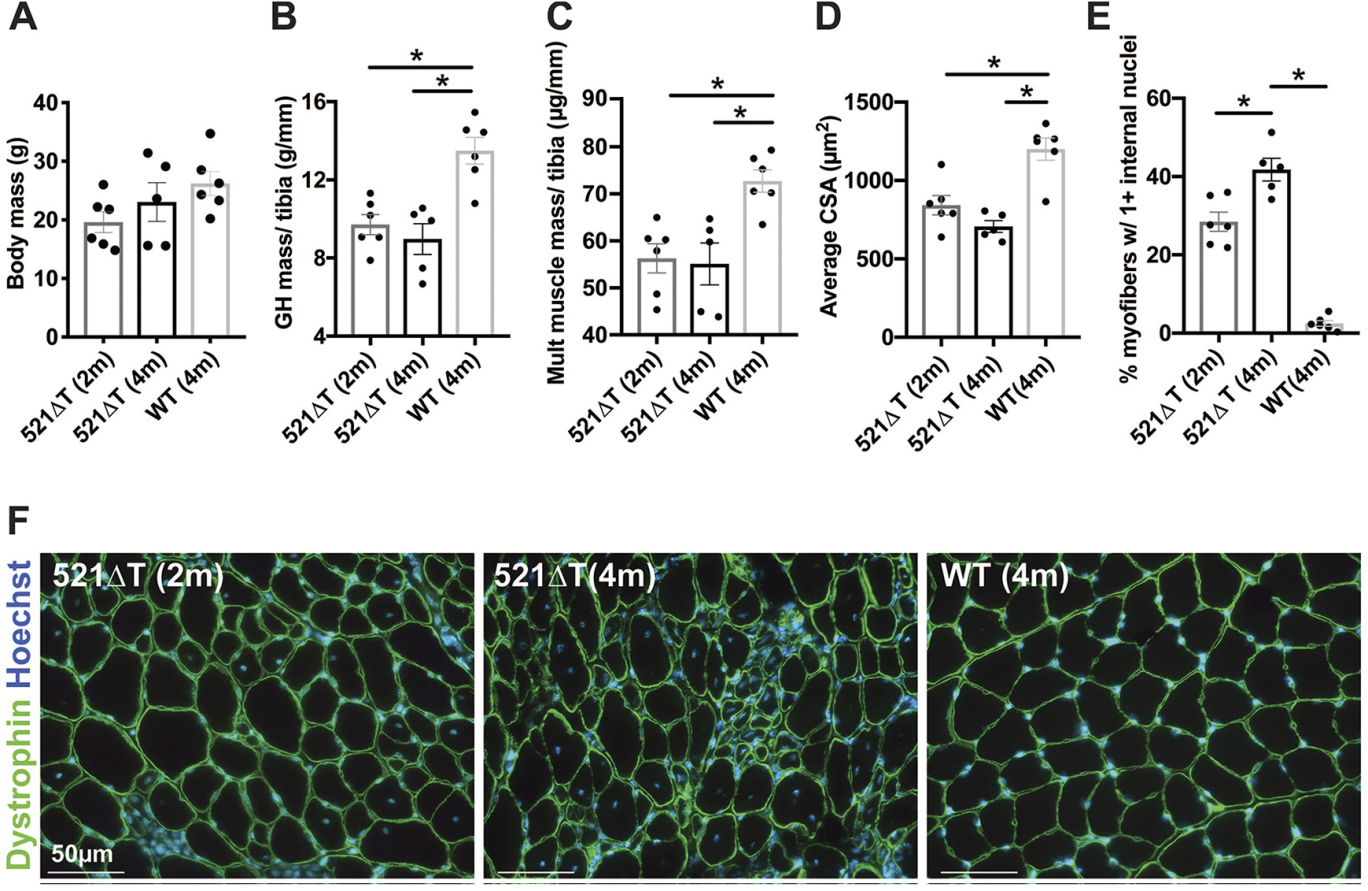
**Reduced mass and myofiber area in *Sgcg* 521ΔT mice.** (A) Body mass was not significantly different among genotypes. (B) Gluteus/hamstring (GH) muscle mass normalized to tibia length was reduced in 521ΔT mice compared with controls. (C) The total mass from muscle groups normalized to tibia length was significantly reduced in 521ΔT mice compared with WT controls (quadriceps, gluteus/hamstring, triceps, diaphragm, heart, tibialis anterior muscle groups combined). (D) Average myofiber cross-sectional area (CSA) was significantly smaller in 521ΔT mice than in WT controls. (E) Percentage of myofibers with >1 internal nuclei was increased in 521ΔT muscle, with 4-month-old 521ΔT myofibers having the most internal nuclei. (F) Representative images of 521ΔT and WT muscle. Anti-dystrophin (green) outlined myofibers. Hoechst (blue) demonstrated nuclei. Data are mean±s.e.m. *P<0.05 (one-way ANOVA with Tukey; *n*≥5 mice per group). Scale bars: 50 µm.

To evaluate the effect of loss of γ-sarcoglycan, mice were further characterized by monitoring total body mass and individual muscle mass. No significant difference in body mass was detected between 521ΔT mice at 2 months or 4 months of age compared with WT controls (Fig. 3A). Examination of gluteus/hamstring (GH) muscle mass normalized to tibia length revealed a reduction in muscle mass in 521ΔT mice compared with WT controls (Fig. 3B). Compilation of muscle mass from multiple muscle groups (quadriceps, gluteus/hamstring, triceps, diaphragm, heart, tibialis anterior combined) normalized to tibia length also revealed a reduction in muscle mass in 521ΔT mice compared with WT controls (Fig. 3C). Correspondingly, the average cross-sectional area of 521ΔT myofibers was reduced compared with WT control myofibers, measured using anti-dystrophin immunofluorescence staining to demarcate the sarcolemma (Fig. 3D,F). Fluorescence imaging also revealed that a greater percentage of 521ΔT myofibers had increased internal myonuclei, at both 2 months and 4 months of age, compared with WT controls (Fig. 3E,F).

**Fig. 4.**
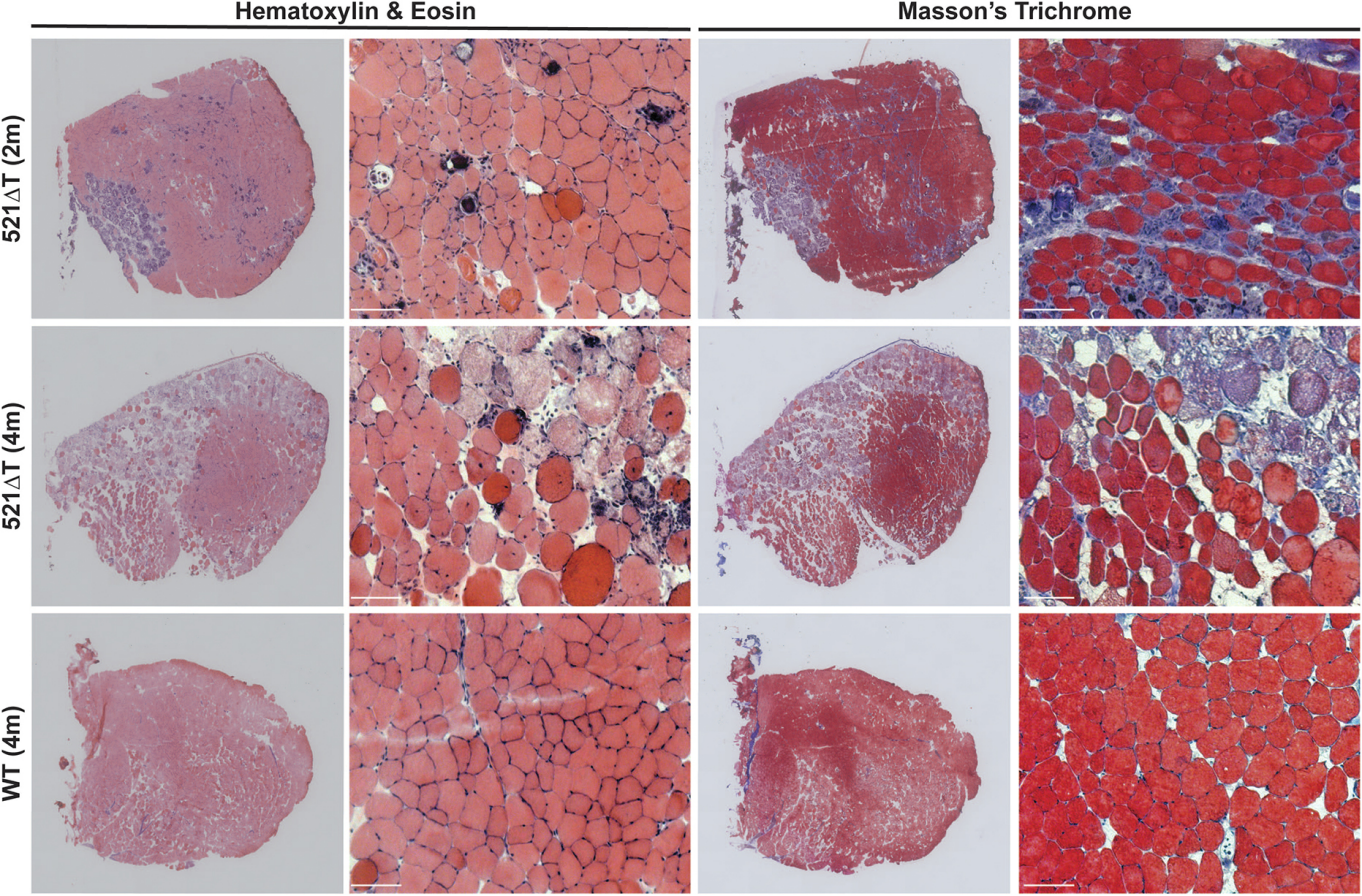
**Marked dystrophic histopathology in *Sgcg* 521ΔT mice.** Tibialis anterior muscle from 521ΔT mice displays hallmark signs of muscle disease including increased immune infiltrate, fibrosis, necrosis, calcification and internal myonuclei visualized through H&E and Masson's Trichrome staining. Older 521ΔT mice (4 months) have more severe muscle pathology than younger 521ΔT (2 months) mice. These features are absent in healthy WT muscle. Scale bars: 100 µm.

**Fig. 5.**
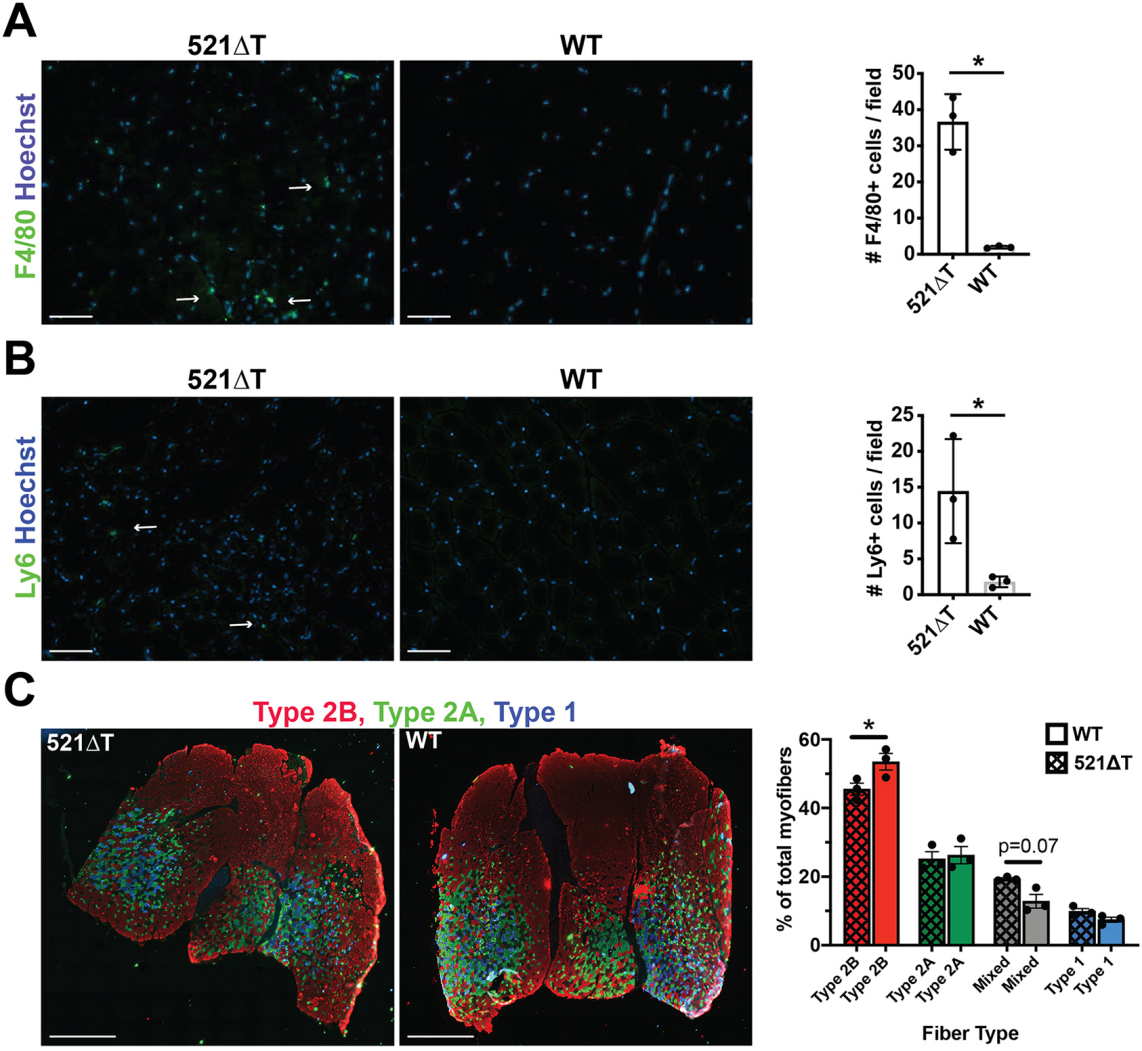
**Increased immune infiltrate and fiber type switching in *Sgcg* 521ΔT muscle.** (A) Anti-F4/80 immunofluorescence staining (green) of gastrocnemius muscle from 4-month-old mice revealed an increase in the average number of F4/80+ macrophages per field in 521ΔT muscle compared with WT controls. Hoechst (blue) labels nuclei. Arrows indicate F4/80+ macrophages. (B) Anti-Ly6 immunofluorescence staining (green) of gastrocnemius muscle from 4-month-old mice revealed an increase in the average number of Ly6+ monocytes/neutrophils in 521ΔT muscle per field. Hoechst (blue) labels nuclei. Arrows indicate Ly6+ monocytes/neutrophils. (C) Representative images and quantification of the fiber type composition of the gastrocnemius muscle from 4-month-old mice, evaluated through immunostaining against the different myosin isoforms. 521ΔT muscle had a significant reduction in the percentage of Type 2B myofibers corresponding with a trend towards an increased number of mixed myofibers (*P*=0.07). Type 2B fibers (red), Type 2A (green), Type 1 (blue), mixed (composed of Type 2B/X, 2X, 2A/X). Data are mean±s.e.m. *P<0.05 (*t*-test in A,B; two-way ANOVA with Bonferroni's correction in C; *n*=3 mice per group). Scale bars: 50 µm (A,B); 1 mm (C).

Histological evaluation through hematoxylin & eosin (H&E) and Masson's Trichrome staining revealed that 521ΔT muscles have muscular dystrophy pathological features, with evidence of increased immune infiltrate, fibrosis, necrosis, calcification, fiber size variability and internal nuclei (Fig. 4). Consistent with enhanced disease progression, histopathological features were more severe in 4-month-old 521ΔT muscle than in 2-month-old 521ΔT tissue (Fig. 4). The extent of immune infiltrate was quantified through immunofluorescence microscopy using an antibody to the macrophage cell surface marker F4/80 and the monocyte/neutrophil marker Ly6. 521ΔT gastrocnemius muscle had a significantly increased number of F4/80+ cells and Ly6+ cells per field compared with WT controls (Fig. 5A,B). In addition, loss of γ-sarcoglycan protein due to the 521ΔT mutation elicited a significant change in fiber type composition, assessed by myosin isoform immunostaining of the gastrocnemius muscle (Fig. 5C). 521ΔT muscle had a significant reduction in the percentage of Type 2B fast glycolytic myofibers, with a trend towards an increased percentage of mixed myofibers (*P*=0.07; 2B/X, 2X, 2A/X combined) compared with WT control muscle (Fig. 5C). These data show that loss of γ-sarcoglycan protein due to the 521ΔT mutation resulted in reduced muscle mass and enhanced muscle pathology.

### Increased muscle leak and fibrosis in 521ΔT mice

**Fig. 6.**
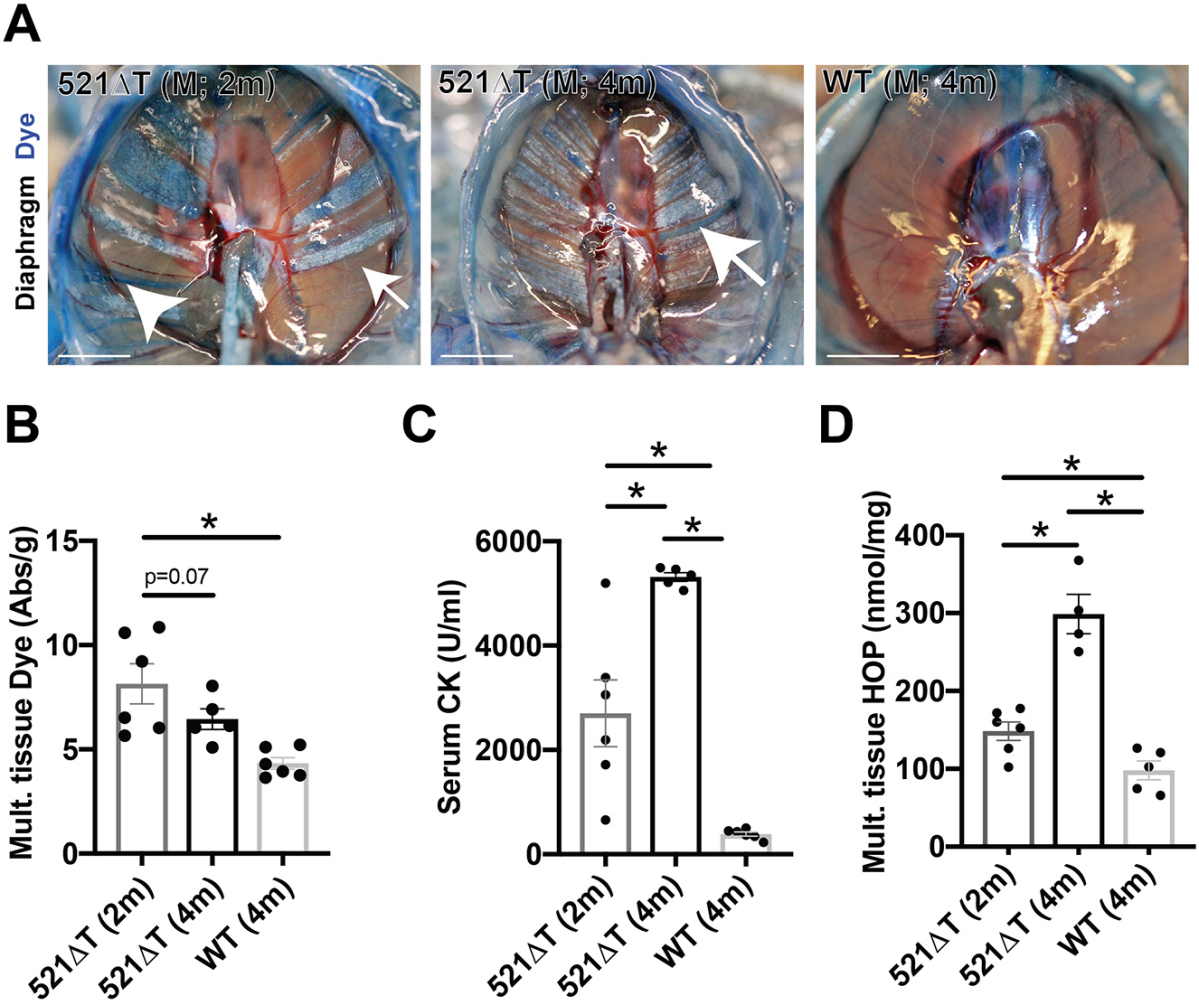
**Increased sarcolemmal membrane leak and fibrosis in *Sgcg* 521ΔT mice.** (A) Mice were injected with Evans Blue dye. Gross imaging shows increased fibrosis (white; arrow) and increased dye uptake (blue streaks; arrowhead) in 521ΔT mice compared with WT controls. (B) Increased dye uptake was seen in 2-month-old 521ΔT muscle by spectrophotometric analysis compared with WT. This increase trended towards a reduction by 4 months of age in 521ΔT muscle (compared with 2-month-old 521ΔT muscle; *P*=0.07). Combined dye values from abdominal, quadriceps, gluteus/hamstring, triceps, diaphragm, heart and gastroc/soleus muscles. (C) Serum CK was elevated in 521ΔT mice compared with controls, with 4-month-old 521ΔT serum CK exceeding that of 2-month-old mutant animals. (D) Fibrosis was measured as HOP content. HOP content was elevated in 521ΔT muscle, with more seen in 4-month-old 521ΔT muscle than at 2 months of age. Combined values from abdominal, gastroc/soleus, diaphragm and triceps. Data are mean±s.e.m. *P<0.05 (one-way ANOVA with Tukey; *n*≥4 mice per group). Scale bars: 5 mm.

Evans Blue dye uptake was evaluated in skeletal muscles from 521ΔT mice as a measure of muscle membrane instability and leak. Evans Blue dye is normally excluded from healthy, intact muscle, but is readily taken up by injured or compromised myofibers (Hack et al., 1998; Matsuda et al., 1995). Gross imaging revealed increased dye uptake in 521ΔT diaphragm muscle compared with WT controls at 2 months and 4 months of age (Fig. 6A). Dye uptake was quantified by spectrophotometric analysis combining values from abdominal, quadriceps, gluteus/hamstring, triceps, diaphragm, heart and gastrocnemius/soleus muscles. Muscle from 2-month-old 521ΔT mice contained significantly more dye than WT controls, and dye uptake in 4-month-old mice showed an increase (Fig. 6B). As a secondary measure of myofiber damage and leak, serum creatine kinase (CK) levels were assessed. CK is highly expressed in skeletal and cardiac muscle, and is released and detected in the serum after injury. Serum CK levels were increased in both 2-month-old and 4-month-old 521ΔT mice compared with WT controls (Fig. 6C). In addition, 4-month-old 521ΔT mice had significantly increased levels of serum CK compared with younger 2-month-old 521ΔT mice (Fig. 6C).

Another pathological feature of LGMD 2C is the progressive replacement of muscle tissue with fibrotic scar (Hack et al., 1998). Gross imaging revealed extensive fibrotic remodeling of 521ΔT tissue (white areas) at 2 months and 4 months of age compared with WT controls (Fig. 6A). Fibrotic content, measured by hydroxyproline (HOP) concentration from multiple muscles (abdominal, gastrocnemius/soleus, diaphragm, triceps combined), were increased in both 521ΔT cohorts compared with WT controls (Fig. 6D) (Smith et al., 2016). HOP content in muscle, reflecting scarring, increased with age, consistent with the progressive nature of the disease (Fig. 6D). These data combined demonstrate that the 521ΔT mice develop progressive myopathy, hallmarked by muscle membrane leak and fibrotic replacement.

### Weakened muscles in Sgcg 521ΔT mice

**Fig. 7.**
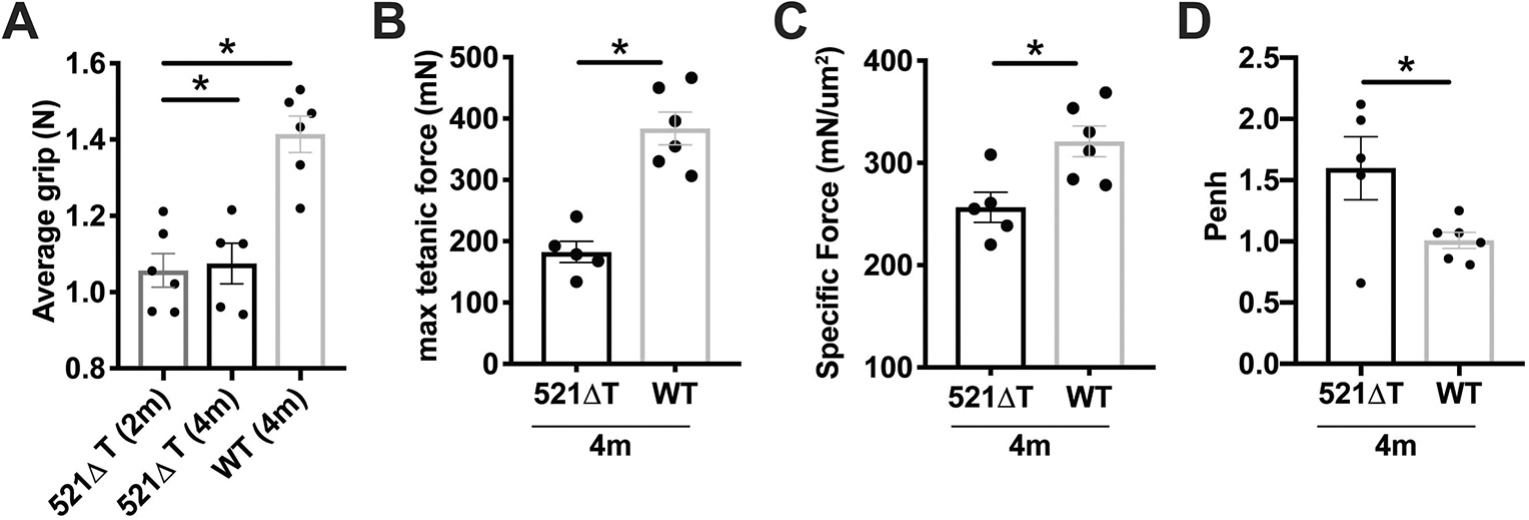
**Reduced muscle function in *Sgcg* 521ΔT mice.** (A) Average forelimb grip strength was decreased in 521ΔT mice. (B) Increased maximum tetanic force was decreased in 521ΔT mice at 4 months of age, measured through *in situ* force analysis of the tibialis anterior muscle. (C) Specific force was also decreased in 521ΔT muscle compared with WT control (tibialis anterior, 4-month-old mice). (D) Respiratory muscle activity was also impaired. Enhanced pause (Penh) was elevated, consistent with abnormal breathing in 521ΔT mice at 4 months, compared with WT controls. Data are mean±s.e.m. *P<0.05 (one-way ANOVA with Tukey in A; *t*-test in B,C,D; *n*≥5 mice per group).

To assess muscle function, muscle force was evaluated. Average forelimb grip strength was significantly decreased in 2-month-old and 4-month-old 521ΔT mice compared with WT controls (Fig. 7A). To further evaluate muscle performance, *in situ* tetanic force of the tibialis anterior muscle was assessed in 4-month-old mice. Maximum tetanic force was significantly decreased in 521ΔT mice compared with age-matched WT controls (Fig. 7B). Moreover, specific force, calculated as tetanic force normalized to muscle cross-sectional area, was also reduced in 521ΔT mice compared with age-matched WT controls (Fig. 7C).

To assess respiratory function, unanesthetized whole-body plethysmography (WBP) was performed on 4-month-old mice. Enhanced pause (referred to as Penh) is a calculation reflecting the timing and pattern of breathing [Penh=(PIP/PEP)×Pause] (PIP, peak inspiratory pressure; PEP, peak expiratory pressure) (Adler et al., 2004). Higher Penh values are indicative of abnormal breathing, and Penh values were increased in 521ΔT mice compared with control (Fig. 7D). When Penh was normalized to body mass, 521ΔT mice trended towards increased values (*P*=0.09; Fig. S3). Additional plethysmography parameters including minute volume/body mass, peak inspiratory flow (PIF)/body mass, peak expiratory flow (PEF)/body mass, and pause were not significantly different between genotypes, whereas time relaxation (Tr) was trending towards significance at *P*=0.09 (Fig. S3). Cardiac function was assessed using echocardiography, and percent fractional shortening was not significantly different than age-matched controls at 4 months of age, despite severe ventricular fibrosis visualized through gross imaging (Fig. S4, bottom panels). Of note, ventricular fibrosis was visible in a subset of WT mice on the DBA/2J background at 4 months of age (Fig. S4, top panels). These data combined demonstrate that at 4 months of age, 521ΔT mice had reduced limb muscle function, and cardiopulmonary function showed early signs of decline.

### *In vivo* multi-exon skipping in 521ΔT muscle

**Fig. 8.**
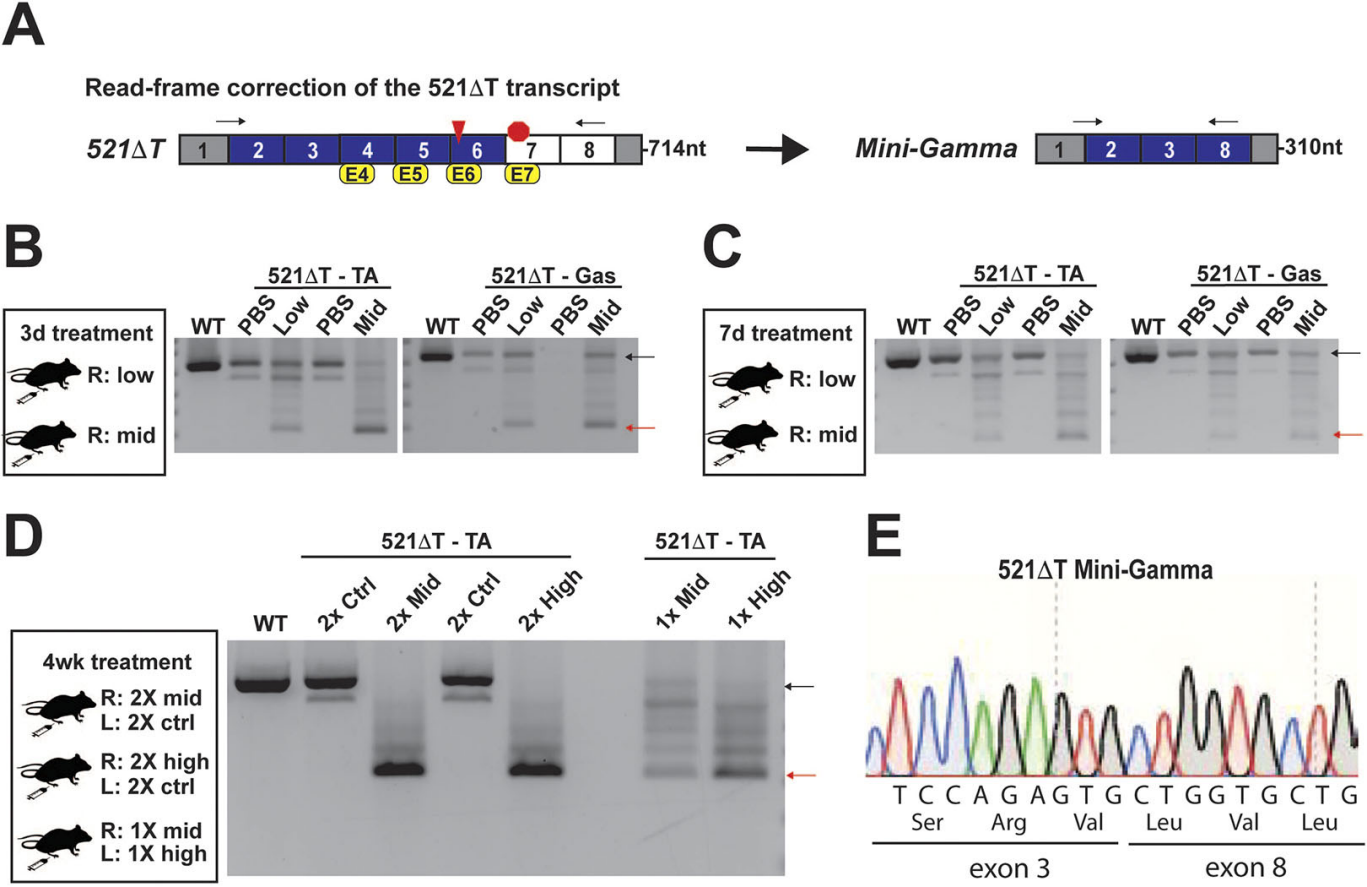
***In vivo* exon skipping in *Sgcg* 521ΔT muscle via IM injection of antisense oligonucleotides.** (A) Schematic showing the correction of the 521ΔT frameshift mutation (red triangle) with a multi-AON exon-skipping strategy targeting exons 4, 5, 6 and 7 (yellow boxes). This strategy corrects the reading frame by generating a transcript encoding Mini-Gamma, comprising exons 2, 3 and 8. The arrows depict the location of the PCR primers, and the expected amplicon size is designated. (B) Design and results from *in vivo* study 1, in which analysis was conducted 3 days after IM injections of AONs into the tibialis anterior (TA) and gastrocnemius/soleus (Gas) muscle. Muscles were injected with either the four-AON cocktail or vehicle control (PBS; ctrl). Gel electrophoresis of RT-PCR products from treated muscles demonstrated the 521ΔT transcript in PBS-treated muscle (black arrow), as well as a dose-dependent generation of the predicted transcript encoding Mini-Gamma in muscle treated with the Mini-Gamma vivo-PMO cocktail at a low and mid dose (red arrow). (C) Similar treatment of two additional mice for 7 days demonstrated similar results but suggested the need for higher AON dosing. (D) Design and results from *in vivo* study 2. RT-PCR results indicate robust generation of the transcript encoding Mini-Gamma after two injections, two weeks apart at both the mid and high doses (red arrow). A concomitant reduction in the unskipped 521ΔT was also seen with both these exposures (black arrow). Mini-Gamma expression was evident 4 weeks after a single mid- or high-dose injection. However, expression was diminished compared with the two-injection treatment, and there were more intermediate products. (E) Gel extraction and subsequent Sanger sequencing confirmed that this product was in-frame Mini-Gamma.

To assess the ability to correct the reading frame of the 521ΔT transcript *in vivo*, AONs were designed to murine *Sgcg* exons 4, 5, 6 and 7 (Fig. 8A). AONs were designed complementary to the splice acceptor regions of *Sgcg* exons 4, 5, 6 and an intraexonic region in exon 7 (Table 1). Chemical modifications of AONs promote stability, enhance RNA binding and prevent RNAse H degradation (Chan et al., 2006). For these studies, we used phosphorodiamidate morpholino oligomers, which contained the vivo-modification for enhanced cell delivery (vivo-PMOs) (Morcos et al., 2008). Individual AONs were resuspended in Dulbecco's phosphate-buffered saline (DPBS), and then combined in a four-AON cocktail on the day of treatment (Tables S1 and S2). AONs were administered via intramuscular (IM) injection, as indicated, and their ability to correct the transcript reading frame was assessed via RT-PCR.

**Table 1.**
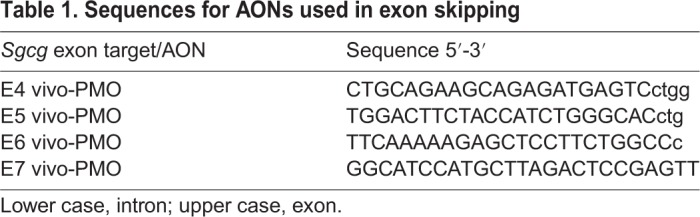
**Sequences for AONs used in exon skipping**

Study 1 was conducted in four 9-week-old 521ΔT mice (Fig. 8B,C). Briefly, the right tibialis anterior and gastrocnemius/soleus muscles were each injected with a four-AON cocktail as detailed in Table S1. The contralateral tibialis anterior and gastrocnemius/soleus muscles from the same mice were injected with an equal volume of vehicle. Muscles from the first set of mice were analyzed 3 days after IM injection, and those from the second set were evaluated 7 days after IM injection. RT-PCR analysis for the *Sgcg* transcript demonstrated expression of the 521ΔT transcript and the endogenous skipping of exon 7 in muscle treated with vehicle alone. A dose-dependent generation of the predicted transcript encoding Mini-Gamma was seen in muscles injected with the four-AON multi-exon skipping cocktail (Fig. 8B,C). This dose-dependent expression of Mini-Gamma persisted for 7 days after IM injection (Fig. 8C).

Study 2 was initiated in 5-week-old 521ΔT mice and continued for 4 weeks (Fig. 8D). In two of three mice in this study, the tibialis anterior was injected with a mid- or high dose of the four-AON cocktail, whereas the contralateral TA was injected with a control AON (Table S2). These two mice received two rounds of injections, an initial injection (day 0) with a second injection after 2 weeks (day 14), and mice were sacrificed for analysis 28 days after the first injection (Fig. 8D). The third mouse received only an initial injection (day 0) of Mini-Gamma AONs (a single mid-dose in the right TA, and a single high dose in the left TA) and was sacrificed on day 28 post injection. RT-PCR analysis demonstrated that both bi-weekly injection regimens generated robust expression of the transcript encoding Mini-Gamma for both the mid- and high dose (Fig. 8D). However, only the high dose maintained Mini-Gamma expression 4 weeks after a single administration (Fig. 8D). The correct sequence of the transcript was confirmed using Sanger sequencing (Fig. 8E). Collectively, these data show that the Mini-Gamma four-AON cocktail corrected the 521ΔT reading frame, producing the transcript in multiple muscle tissues *in vivo*.

## DISCUSSION

### A new model for testing LGMD therapy

The previous well-characterized mouse model for LGMD 2C was useful to elucidate basic mechanisms of sarcolemmal instability and for testing gene replacement therapy (Cordier et al., 2000; Hack et al., 2000, 1998). This previous model was also useful for showing that transgenic expression of Mini-Gamma protein was functional and could alleviate the dystrophic phenotype (Gao et al., 2015). Although significant progress is being made using viral gene replacement therapy, current approaches are limited by neutralizing antibodies and potential immune response to the expressed protein. At the same time, genetic correction using AON-meditated exon skipping has received approval from the FDA for the treatment of DMD and for spinal muscular atrophy (Aartsma-Rus and Krieg, 2017). Previous studies using a four-AON cocktail were piloted using patient-derived human cell lines, including an LGMD 2C patient carrying the most common 521ΔT mutation (Wyatt et al., 2018). In the previous studies, vivo-PMOs appeared to have better efficacy for skipping, although this likely relates to modifications to improve AON access into cells. Despite this success, an appropriate *in vivo* model to test this therapeutic was lacking. We have now used CRISPR/Cas9 gene editing to develop a precision model of LGMD 2C. This model demonstrated a dystrophic phenotype and muscle pathology similar to that observed in patients with the most severe forms of the disease and generates a new platform that is genetically appropriate for testing multi-exon skipping.

### Challenges for *in vivo* studies

Transgenic rescue of the dystrophic phenotype in *Sgcg-*null mice used a muscle-specific transgene to overexpress the Mini-Gamma protein compared with full-length WT protein (Gao et al., 2015). This earlier study used *Sgcg* mice in the C57BL/6J background, which is known to have a milder phenotype than those in the DBA/2J background (Heydemann et al., 2005). The current study created the 521ΔT mutation on the C57BL/6J background and then backcrossed to the DBA2/J background, ensuring the presence of the severe homozygous *Ltbp4* allele (Heydemann et al., 2009). The *Ltbp4* allele contributes significantly to generating increased fibrosis and membrane leak, contributing up to 40% of the phenotypic variance (Swaggart et al., 2014). 521ΔT mice on the C57BL/6J background were not extensively bred or phenotyped, limiting comparisons of phenotypic differences between the DBA/2J and C57BL/6J strains. This 521ΔT DBA/2J model demonstrates reduced muscle mass, increased fibrosis and muscle membrane leak, similar to that seen when dystrophin gene mutations are in the DBA/2J background (Coley et al., 2016). We did not observe a reduction in left ventricular cardiac function, and we expect that this relates to the relatively young age of the mice, as *Sgcg*-null mice in this same DBA/2J background demonstrate cardiopulmonary decline at 6 months of age (Quattrocelli et al., 2017b). However, the trend toward decline in respiratory parameters and evidence for cardiac fibrosis at 4 months of age can be viewed as earlier indicators that are expected to progress, mirroring the progression that is observed in human patients.

We have now used this model to conduct pilot experiments testing AON-mediated exon skipping directly in muscle. The complexity of multi-exon skipping relies on AONs being taken up into individual myofibers. Exon skipping studies in a canine DMD model suggest that multi-exon skipping is feasible (Lim et al., 2019). Herein, we show successful generation of the transcript encoding Mini-Gamma in skeletal muscle *in vivo*. Evaluating multi exon-skipping-induced Mini-Gamma protein expression and function is a critical next step. We expect that systemic, repetitive administration of the AON cocktail will be required to reliably detect protein expression. In addition, optimization of dosage for each AON will be required to determine the optimal regimen that elicits Mini-Gamma protein expression and ultimately pathological and functional benefit in the 521ΔT model. It is also likely that AON sequence optimization will impact the degree of Mini-Gamma production. The current AON sequences predominately target the exon splice acceptor sites; however, efficient skipping has been observed using intraexonic sequences that target exon splicing elements (Aartsma-Rus and van Ommen, 2007). Although there are some differences between the human and mouse intraexonic sequences, the location of these putative sites is similar relative to the exon start site in both species.

### Broader applications for the 521ΔT model

The generation of an LGMD 2C model through the introduction of the most common 521ΔT mutation will have broader applications. This is one of the few models that requires multi-exon skipping to achieve gene correction and enables better assessment of this potential. Although this is more difficult to execute systemically, it allows for more potential therapeutic targets that can impact larger populations of patients (Echigoya and Yokota, 2014). Furthermore, new chemistries are in development to improve delivery and reduce dosage. These include the tricyclo-AON modification and the use of ultrapure stereoisomers of AON compounds (Goyenvalle et al., 2015; Iwamoto et al., 2017). This model allows not only the study of LGMD 2C, but also the interrogation of these systems in a multi-exon skipping setting. Finally, this model will allow the potential use of gene editing with CRISPR to correct the same mutation this system introduced, paving the way for the next generation of gene therapies.

## MATERIALS AND METHODS

### Gene-edited zygotes

Mice harboring the *Sgcg* exon 6 521ΔT single thymine deletion mutation were generated through the Northwestern University Transgenic and Targeted Mutagenesis Laboratory (TTML). Briefly, gRNA targeting the locus were identified using the CRISPR Design Tool (crispr.mit.edu). The optimal gRNA was identified on the anti-sense strand with the sequence 5′-TGTTCAAAAAGAGCTCCTTCTGG-3′. The gRNA complex was synthesized using the GeneArt Precision gRNA Synthesis Kit (A29377, Thermo Fisher Scientific). Cas9 mRNA was purchased from Thermo Fisher Scientific (GeneArt A29378). The 200 nt single-stranded (ssODN) ultramer repair template, harboring the single thymine deletion, was synthesized and PAGE purified by Integrated DNA Technologies. It was designed towards the antisense strand as follows 5′-atgagaacgtttgtttatattgagtaaattcttacCTAAGGTCTTGAAATGGGTCAGCTCTGACAAGGGGTGTCTCCACTGAGTGTTCAAAAGAGCTCCTTCTGGCCctaaaagaagaccagagatgtgtcagagacaaaagtacaagaagtagcctcagccaaactaacagacagtcca-3′ (upper case indicates exonic sequence; lower case indicates intronic sequence). All CRISPR components were microinjected into the cytoplasm of zygotes.

### Mouse generation

Mice were bred and housed in a specific pathogen-free facility on a 12-h light/dark cycle and fed *ad libitum* in accordance with and under the approval of the Northwestern University Institutional Animal Care and Use Committee. After pronuclear injection, embryos were transferred into pseudo-pregnant C57BL/6J mice. Tail DNA was isolated from 13 potential founders using the Qiagen PureGene Tissue Isolation Kit (158667, Qiagen). Tail DNA was genotyped using the following primer set flanking *Sgcg* exon 6: primer SAF 5′-TGTACAAAAAAGCAGGCTTTAAAG-3′ and primer SAR 5′-TAATGCCAACTTTGTACAAGAAAG-3′. PCR products were Sanger sequenced using the same primer set, and examined for the presence of the 521ΔT point mutation. PCR products from three potential founders were cloned into the TOPO p2.1 cloning vector (K450001, Thermo Fisher Scientific) and analyzed for the presence of the 521ΔT allele. A single male with a heterozygous 521ΔT mutation was bred with a WT C57BL/6J female. The heterozygous F1 offspring was then backcrossed into the DBA/2J mouse strain for five generations to generate a more severe dystrophic phenotype (Fig. S1). These mice were genotyped for the deleterious *Ltbp4* allele, as described in Heydemann et al. (2009) and Swaggart et al. (2014).

### 521ΔT characterization

Muscle was dissected from 521ΔT D2BA/2J F5 mice bred to homozygosity along with the muscle from WT littermates. Muscle was flash frozen in liquid nitrogen. Total RNA was isolated from the tibialis anterior as described below, and analyzed for the expression of the *Sgcg* and 521ΔT transcripts using the following primer sets: F, 5′-ACTCACATAGAGAGGCCCGA-3′; R, 5′-CCATCAGGACACGCACAGAT-3′. Individual bands were gel extracted, purified using the Qiaex 2 Gel Extraction Kit (20021, Qiagen) and submitted for Sanger sequencing. Extracted PCR products were also subcloned into the TOPO PCR 2.1 vector described above, mini-prepped and submitted for Sanger sequencing. For detection of γ-sarcoglycan, 10 µm muscle sections were fixed for 2 min in ice-cold methanol. After fixation, muscle was rinsed with PBS, blocked in PBS plus 10% fetal bovine serum and 0.1% Triton X-100 (9002-93-1, Sigma-Aldrich) for 1 h at 4°C, then incubated with anti-γ-sarcoglycan antibody (1:250; McNally, 1996a). Sections were rinsed and incubated with goat anti-rabbit IgG H&L Alexa-Fluor-594 secondary antibody (150080, Abcam, 1:2500) and Hoechst 33342 (H3570, Thermo Fisher Scientific, 1:10,000) for 1 h at room temperature. Sections were fixed in Vectashield mounting media (H-1000, Vector Laboratories) and imaged on a Zeiss Observer microscope using Zen Pro software.

### RNA isolation

Fresh or flash-frozen muscle was directly added to 1 ml of Trizol reagent (15596026, Thermo Fisher Scientific) containing zirconia/silica beads (11079125z, BioSpec Products). The samples were homogenized for 1 min using a BioSpec bead beater. After homogenization, samples were spun at 12,000 ***g*** for 5 min at 4°C. Then 900 µl of supernatant was transferred to 1.5 ml tubes, 200 μl of chloroform was added to the samples, shaken and incubated for 5 min at room temperature. Samples were then spun at 12,000 ***g*** for 15 min at 4°C. The supernatant was then transferred to a fresh 1.5 ml tube and further processed using the Aurum RNA Isolation Kit (7326820, Bio-Rad Laboratories). Finally, 1 µg of RNA was reverse-transcribed to cDNA using the qScript reagent (95048, Quantabio).

### Immunoblotting

Muscles were dissected and lysed in whole-tissue lysis buffer [50 mM HEPES (pH 7.5), 150 mM NaCl, 2 mM EDTA, 10 mM NaF, 10 mM Na-pyrophosphate, 10% glycerol, 1% Triton X-100, 1 mM phenyl-methylsulfonyl fluoride (PMSF), 1× cOmplete™ Protease Inhibitor Cocktail (11697498001 CO-RO, Roche)] using a glass dounce tissue grinder. The protein concentration of the muscle or cell lysate was determined using the Quick Start™ Bradford Protein Assay (5000205, Bio-Rad Laboratories). Proteins were separated on a 10% Mini-Protean^®^ TGX™ Precast Protein Gel, 15-well, 15 µl (4561036, Bio-Rad Laboratories) and transferred to Immun-Blot PVDF Membranes for protein blotting (1620177, Bio-Rad Laboratories). Blocking and antibody incubations were carried out using StartingBlock T20 (TBS) Blocking Buffer (37543, Thermo Fisher Scientific). The primary antibody used was Novocastra^TM^ lyophilized mouse monoclonal antibody gamma-sarcoglycan (NCL-g-SARC, Leica Biosystems). Goat anti-mouse secondary antibody conjugated to horseradish peroxidase (115-035-003; Jackson ImmunoResearch) was used at a dilution of 1:2500. SuperSignal™ West Pico Chemiluminescent Substrate and SuperSignal™ West Femto Maximum Sensitivity Substrate (34080 and 34096, respectively, Thermo Fisher Scientific) were applied to membranes and membranes were visualized using an Invitrogen™ iBright™ CL1000 imaging system (A32749; Thermo Fisher Scientific). Pierce™ Reversible Protein Stain Kit for PVDF Membranes (24585, Thermo Fisher Scientific) was used to stain the blot to ensure complete transfer and equal loading. Immunoblot bands were quantified using FIJI gel analysis tools.

### Immunofluorescence microscopy

For detection of dystrophin, 10 µm muscle sections were fixed with 4% PFA made from 16% paraformaldehyde solution (15710, Electron Microscopy Sciences). After fixation, muscle was rinsed with PBS, blocked in PBS plus 10% fetal bovine serum and 0.1% Triton X-100 (9002-93-1; Sigma-Aldrich) for 1 h at 4C, then incubated with anti-dystrophin primary antibody (PA1-37587, Thermo Fisher Scientific) at 1:100 overnight at 4°C. Sections were rinsed and then secondary goat anti-rabbit IgG H&L Alexa-Fluor-488 (150077, Abcam, 1:2500) and Hoechst (1:10,000) were used for 1 h at room temperature. All samples were fixed in ProLong^®^ Gold Antifade Mountant (P36930; Thermo Fisher Scientific) and imaged on a Zeiss Observer microscope using Zen Pro software.

Immunofluorescence staining to evaluate fiber type was performed as described below. Frozen muscle sections (10 µm) of the gastrocnemius muscle were fixed in ice-cold acetone for 5 min, rinsed in PBS and then blocked in 1% bovine serum albumin and 10% fetal bovine serum in PBS for 1 h. For fiber typing, sections were incubated with primary antibodies BA-F8 (1:10), SC-71 (1:30) and BF-F3 (1:10), all from the Developmental Studies Hybridoma Bank, overnight at 4°C. Sections were rinsed in PBS plus 1% bovine serum albumin and then incubated with secondary antibodies Alexa-Fluor-350 anti-IgG2b, Alexa-Fluor-488 anti-IgG1, and Alexa-Fluor-594 anti-IgM (A21140, A21121 and 1010111, respectively, Life Technologies; all used at 1:500) for 1 h. Following secondary incubation, sections were again rinsed in PBS plus 1% bovine serum albumin. All primary and secondary antibodies were diluted in a 1% bovine serum albumin PBS solution. All steps were carried out at room temperature using room temperature reagents, except where noted. All samples were fixed in ProLong^®^ Gold Antifade Mountant (P36930; Thermo Fisher Scientific) and imaged on a Zeiss Observer microscope using Zen Pro software. Type 2B, Mixed (2B/X, 2X, 2X/A combined), Type 2A and Type 1 fibers were quantified and expressed as the % of total myofibers per muscle section.

For immune cell detection, sections were fixed in formalin for 10 min, then rinsed in PBS. Sections were blocked with 1% bovine serum albumin and 10% fetal bovine serum (F4/80) or 0.1% Triton X-100 and 10% fetal bovine serum (Ly6). Following blocking, sections were incubated with a combination of anti-dystrophin at a dilution of 1:100 and either anti-F4/80 conjugated to Alexa-Fluor-488 (ab6640; Abcam, 1:100) or anti-Ly6 conjugated to FITC (553126; BD Biosciences, 1:100) overnight at 4°C. Sections were rinsed in cold PBS, following which sections were incubated with secondary goat anti-rabbit IgG H&L Alexa-Fluor-594 (A-11012; Invitrogen, 1:2500) and Hoechst (1:10,000) for 1 h at 4°C. All steps were carried out on ice using cold reagents. All samples were fixed in ProLong Gold Antifade Mountant (P36930; Thermo Fisher Scientific) and imaged on a Zeiss Observer microscope using Zen Pro software. Immune cell number was quantified from at least three fields per muscle, from *n*=3 mice per genotype, at 4 m of age. Data is expressed as the average number of positive cells per field.

### Physiologic analyses

#### Body weight and muscle analysis

Total body mass was determined and, after sacrifice, tibia lengths were measured. Raw body mass was normalized to the average tibia length. Muscles were removed and immediately weighed. Muscle mass was normalized to tibia length. Excised muscles were immediately frozen in liquid nitrogen, placed in pre-cooled Nalgene cryovials and stored at −80°C or placed in Fisher HealthCare™ Protocol™ 10% buffered formalin (23-305510, Thermo Fisher Scientific) for processing.

#### Grip strength

Grip strength assessments were performed using a Chantillon Ametek Force Transducer in a Columbus Instruments apparatus. Mice were held firmly by the tail, allowed to grasp the attached triangular bar equally with both forepaws, and then pulled away horizontally until the grip was released. Force was recorded for 10 consecutive pulls separated by 15 s. Values are reported as the average of the trials and as average force normalized to whole body weight. The same operator performed all assessments and was blinded to genotype.

#### Plethysmography

Unanesthetized WBP was used to measure respiratory function using a Data Sciences International, Buxco Finepointe 4-site WBP as described (Quattrocelli et al., 2017a). Individual mice were placed in a calibrated cylindrical chamber. Each mouse was allowed to acclimate to the plethysmography chamber for 120 min before recording was initiated. Mice were generally resting during acquisition. Data was recorded for a total of 20 min. Studies were performed at room temperature. Data analysis included breaths with a 0 Rejection Index (Rinx) and in a frequency range of 100-250 breaths per minute. Data was also normalized to body mass (g) where appropriate.

#### Serum collection

Serum was acquired and processed as described (Demonbreun et al., 2016). Briefly, blood was collected by means of retro-orbital puncture with heparinized capillary tubes (20-362-566, Thermo Fisher Scientific) into a Microtainer™ Gold Top Serum Separator (365967, Becton Dickinson) and centrifuged at 8000 ***g*** for 10 min. The plasma fractions were frozen and stored at −80°C.

#### Serum biomarkers

Serum CK was analyzed in triplicate for each mouse using the EnzyChrom Creatine Kinase Assay (ECPK-100, BioAssay Systems) following the manufacturer's instructions. Results were acquired with the Synergy HTX multi-mode plate reader (BioTek^®^).

#### Echocardiography

*In vivo* cardiac function was assessed by echocardiography and was conducted under anesthesia (0.8 liters/min, 1% vaporized isoflurane in 100% O_2_) as described previously (Barefield et al., 2017). Echocardiography was performed using a Visual Sonics Vevo 2100 imaging system with a MS550D 22-55 MHz solid-state transducer (FujiFilm). Cardiac function was assessed using short-axis M-mode to measure left ventricular wall thickness and chamber diameter. Heart rate was maintained >450 beats per minute to minimize variability. Percent fractional shortening was calculated as: 100×[(LV diastolic diameter−LV systolic diameter)/LV diastolic diameter].

#### *In situ* force and fatigue

Muscle mechanics were performed as described previously (Quattrocelli et al., 2017a). Briefly, immediately before sacrifice, *in situ* tetanic force from tibialis anterior muscle was measured using a Whole Mouse Test System (#1300A, Aurora Scientific) with a 1 N dual-action lever arm force transducer (300C-LR, Aurora Scientific) in anesthetized animals (0.8 liters/min of 1.5% isoflurane in 100% O_2_). Both muscles per animal were assayed. Tetanic isometric contraction was induced with following specifications: initial delay, 0.1 s; frequency, 200 Hz; pulse width, 0.5 ms; duration, 0.5 s; using 100 mA stimulation. Length was adjusted to a fixed baseline of 50 mN resting tension for all muscles/conditions. Specific force was calculated as tetanic force normalized to muscle cross-sectional area assayed for each muscle from every animal. Fatigue analysis was conducted by repeating tetanic contractions every 10 s until complete exhaustion of the muscle (25 cycles). Time of contraction was assessed as time to maximum tetanic value within the 0.0-0.5 s range of each tetanic contraction, and time of relaxation was assessed as time to 90% minimum tetanic value within the 0.5-0.8 s range of every tetanus.

### Evans Blue dye uptake quantification

Evans Blue dye uptake was measured as described previously (Heydemann et al., 2009; Thurston et al., 1999). Mice were injected intraperitoneally with 5 µl/g of 10 µM Evans Blue dye (E2129, Sigma-Aldrich). Mice were sacrificed ∼24 h post injection. Multiple muscle groups were assessed including the abdominal, diaphragm, quadriceps, gastrocnemius/soleus, gluteus/hamstrings and triceps and values normalized to tissue weight and kidney dye uptake. Each sample was assessed in duplicate. Absorbance was measured at 620 nm on a Synergy HTX multi-mode plate reader (BioTek^®^). Results are reported as arbitrary optical density units/mg of tissue.

### Hydroxyproline quantification

The content of hydroxyproline in each tissue was assayed as described previously (Flesch et al., 1997; Heydemann et al., 2009). Skeletal muscle tissues (quadriceps, abdominal, gastrocnemius/soleus, gluteus/hamstrings, diaphragm and triceps) were assayed. A standard curve was created using 0-5000 nMol hydroxyproline (H-5534, Sigma-Aldrich) as starting material and the zero mM standard was used as a blank when measuring absorbance. Each sample was assessed in triplicate. Amount of hydroxyproline in each tissue was normalized to tissue mass, and the results are reported as nMol hydroxyproline/mg tissue.

### Muscle analysis

Muscles were dissected and frozen in liquid nitrogen. Anti-dystrophin sarcolemmal fluorescence outlined individual myofibers and was used to assess the myofiber mean cross-sectional area automatically using FIJI (NIH) from at least five fields per mouse. The percentage of fibers with central nuclei marked by Hoechst fluorescence was calculated from the number of fibers containing internalized nuclei in each image/the total number of fibers counted per image, standardized as a percentage. Images were captured using a Zeiss Axiophot microscope.

### Histology

Excised muscles were placed in 10% formaldehyde (245-684, Thermo Fisher Scientific) for histological processing. Sections (10 μm thick) from the center of paraffin-embedded muscles were stained with H&E (12013B and 1070C, Newcomer Supply) and Masson trichrome (HT-15, Sigma-Aldrich). Images were captured using a Zeiss Axiophot microscope.

### Vivo-PMOs

Vivo-PMOs were designed according to described guidelines and synthesized as 25-mer vivo-PMOs by GeneTools. Specific AON sequences and their exon targets are listed in Table 1. A non-targeting vivo-PMO standard control was purchased from GeneTools (CCTCTTACCTCAGTTACAATTTATA). Vivo-PMO were resuspended in pure DPBS to a stock concentration of 0.5 mM.

### IM injection of vivo-PMO

A four-AON vivo-PMO cocktail was directly injected into the tibialis anterior or gastrocnemius/soleus of 521ΔT mice between the ages of 5 and 9 weeks old. Briefly, the four-AON cocktail was combined in a final volume of 20-40 μl of DPBS as indicated in Table S2. Either DPBS or a non-targeting vivo-PMO was used as a control. Mice were anesthetized using isoflurane. Mice underwent either a single or multi-injection regimen, as detailed in Fig. 8. Total RNA from these samples was isolated, reverse-transcribed, sequenced and analyzed as described above.

### Statistical analysis

Statistical analyses were performed using Prism software v7.0a (Graphpad). When comparing two groups, two-tailed Student's *t*-test with Welch's correction (unequal variances) was used. When comparing three or more groups of data for only one variable, one-way ANOVA with Tukey multi-comparison was used, whereas a two-way ANOVA was used for two variables. *P*-value ≤0.05 was considered significant. Data were presented as single values were appropriate. In analyses pooling larger data point sets per group, Tukey distribution bars were used to emphasize data range distribution. Error bars represent±s.e.m.

This article is part of a special collection ‘A Guide to Using Neuromuscular Disease Models for Basic and Preclinical Studies’, which was launched in a dedicated issue guest edited by Annemieke Aartsma-Rus, Maaike van Putten and James Dowling. See related articles in this collection at http://dmm.biologists.org/collection/neuromuscular.

## Supplementary Material

Supplementary information
