# Supplementary material for: A gene-edited mouse model of limb-girdle muscular dystrophy 2C for testing exon skipping
Source: Dis Model Mech. 2019 Nov 4;13(2):dmm040832. doi: 10.1242/dmm.040832 (PMC6906631; doi:10.1242/dmm.040832)
Supplement: Supplementary information [file dmm-13-040832-s1.pdf]

SUPPLEMENTAL INFORMATION

Table S1. AON dosing for Study 1

AON	Low dose	Mid dose
Exon 4	1.5µg / 0.3µL	4.5µg / 0.9µL
Exon 5	0.5µg / 0.1µL	1.5µg / 0.3µL
Exon 6	2.5µg / 0.6µL	7.5µg / 1.5µL
Exon 7	0.5µg / 0.1µL	1.5µg / 0.3µL
TOTAL	5.0µg / 1.1µL	15.0µg / 3.3µL

AON concentration 5µg/µL; final volume of each reaction was 40µL in PBS

Table S2. AON dosing for Study 2

AON	Mid dose	High dose
Exon 4	4.5µg / 0.9µL	12µg / 2.4µL
Exon 5	1.5µg / 0.3µL	4µg / 0.8µL
Exon 6	7.5µg / 1.5µL	20µg / 4.0µL
Exon 7	1.5µg / 0.3µL	4µg / 0.8µL
TOTAL	15.0µg / 3.3µL	40µg / 8.0µL

AON concentration 5µg/µL; final volume of each reaction was 20µL in PBS

Disease Models & Mechanisms • Supplementary information

A

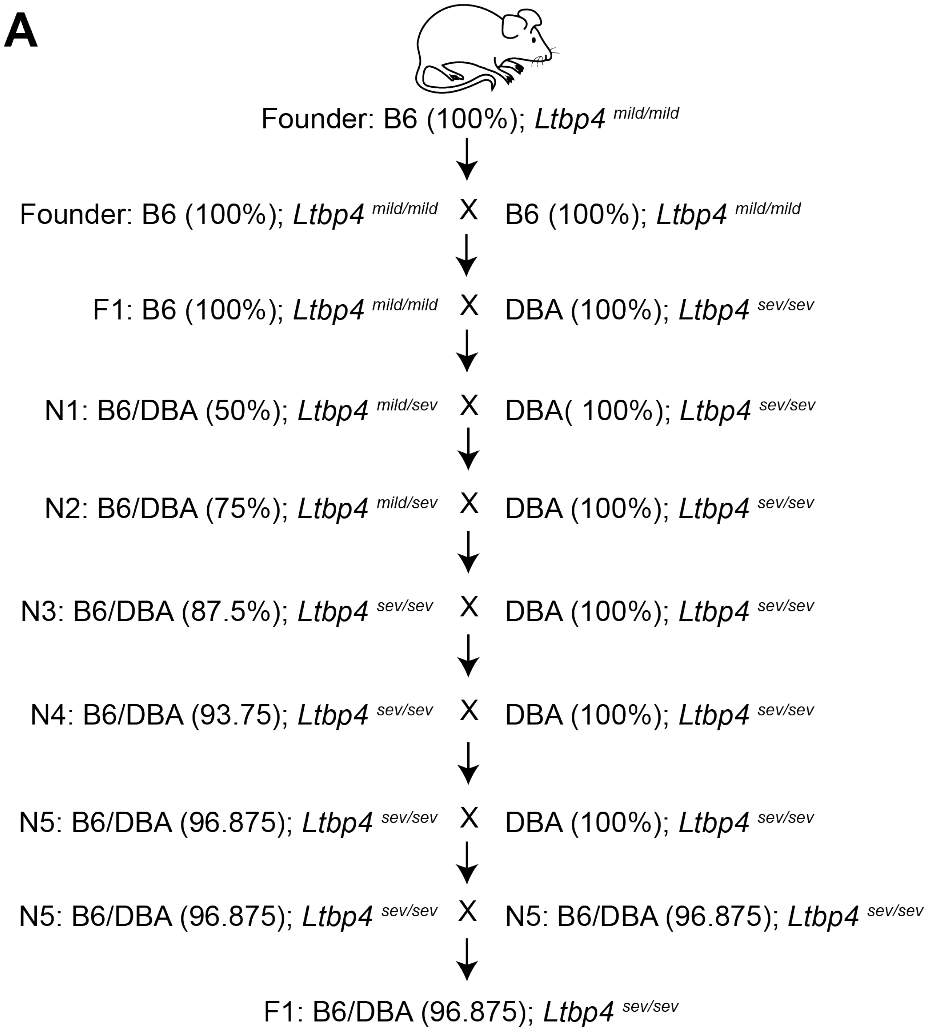

B

	521ΔT +/+	521ΔT +/-	521ΔT -/-	Total
**Born**	20 (24%)	40 (48%)	23 (28%)	83
**Expected**	20.75 (25%)	41.5 (50%)	20.75 (25%)	83

Figure S1. A) Breeding schematic of the 521ΔT mouse model. Founder 521ΔT mice were generated on the C57BL/6J background containing the mild *Ltbp4* allele (*Ltbp4*$^{mild/mild}$). The founder 521ΔT mouse was mated once with a C57BL/6J mouse. An F1 offspring that was 100% C57BL/6J and carried the 521ΔT mutation was backcrossed to the DBA/2J background strain to generate mice that have the 521ΔT mutation and the severe *Ltbp4* allele (*Ltbp4*$^{severe/severe}$). Mice were backcrossed to the DBA/2J strain over 5 generations to generate mice that were >96% DBA, carry the 521ΔT mutation and are homozygous for the severe *Ltbp4* allele. **B)** 521ΔT mice were born in expected ratios.

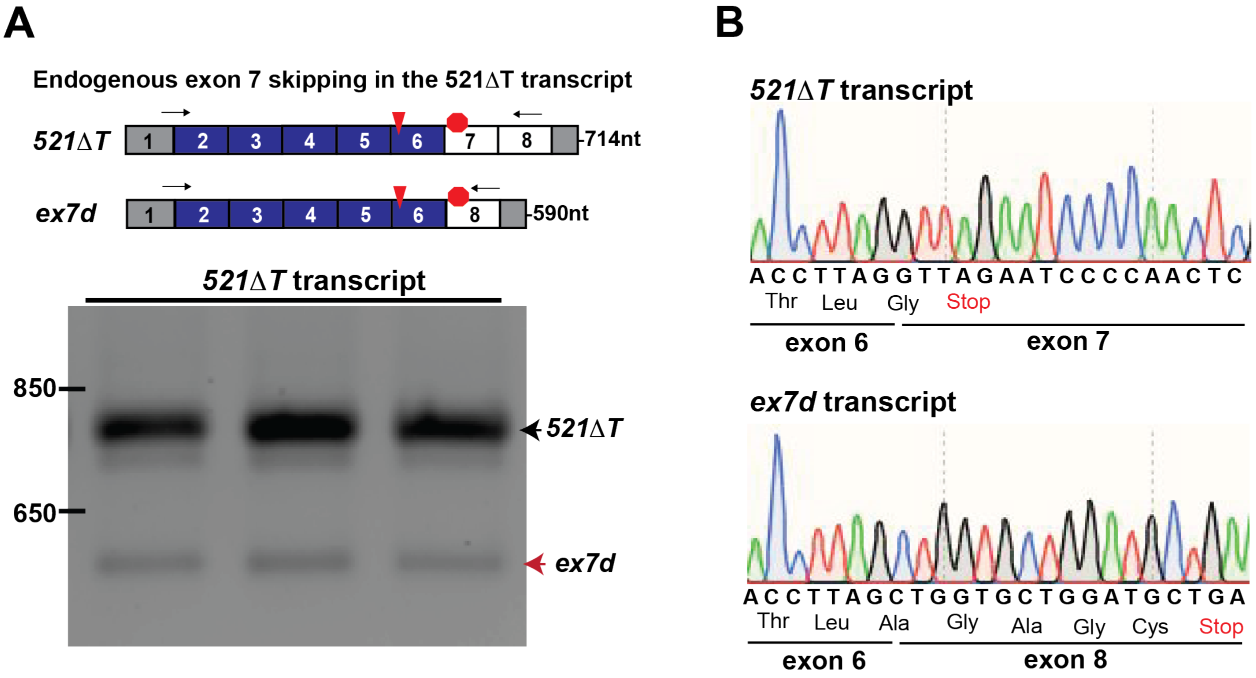

Figure S2. Endogenous skipping of *Sgcg* exon7. A) Schematic showing the effects of the predicted endogenous skipping of exon 7 in the 521ΔT transcript. Like 521ΔT, the skipping exon 7 also generates a premature stop codon and does not result in protein expression. Gel electrophoresis of the *521ΔT* transcript shows a lower band of approximately 590bp that corresponds to the skipping of exon 7. **B)** Gel excision of the bands followed by Sanger sequencing confirms the skipping of exon 7 in the lower band.

Disease Models & Mechanisms • Supplementary information

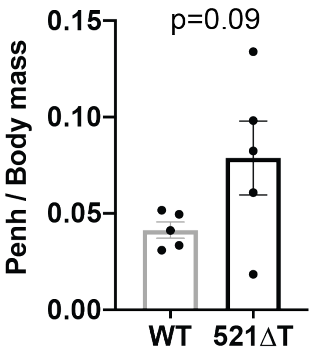

	WT	521ΔT	p-value	n
Freq	154 (±6.8)	158 (±13.1)	0.79	5
Penh/mass	0.041 (±0.004)	0.078 (±0.019)	0.09	5
MV/mass	1.12 (±0.8)	1.2 (±0.7)	0.48	5
PIF/mass	0.084 (±0.009)	0.083 (±0.008)	0.94	5
PEF/mass	0.058 (±0.005)	0.073 (±0.007)	0.12	5
Tr	0.11 (±0.003)	0.099 (±0.006)	0.09	5
Pause	1.3 (±0.02)	1.5 (±0.21)	0.39	5

Figure S3. Whole-body, unanesthetized plethysmography was performed on wildtype and 521ΔT mice at 4 months of age. Enhanced pause (Penh) normalized to body mass was trending towards significance p=0.09. Additional plethysmography parameters including frequency, minute volume, peak inspiratory flow, and pause were not significantly different. Peak expiratory flow and time relaxation were trending towards significance. n=5 mice per group.

Disease Models & Mechanisms • Supplementary information

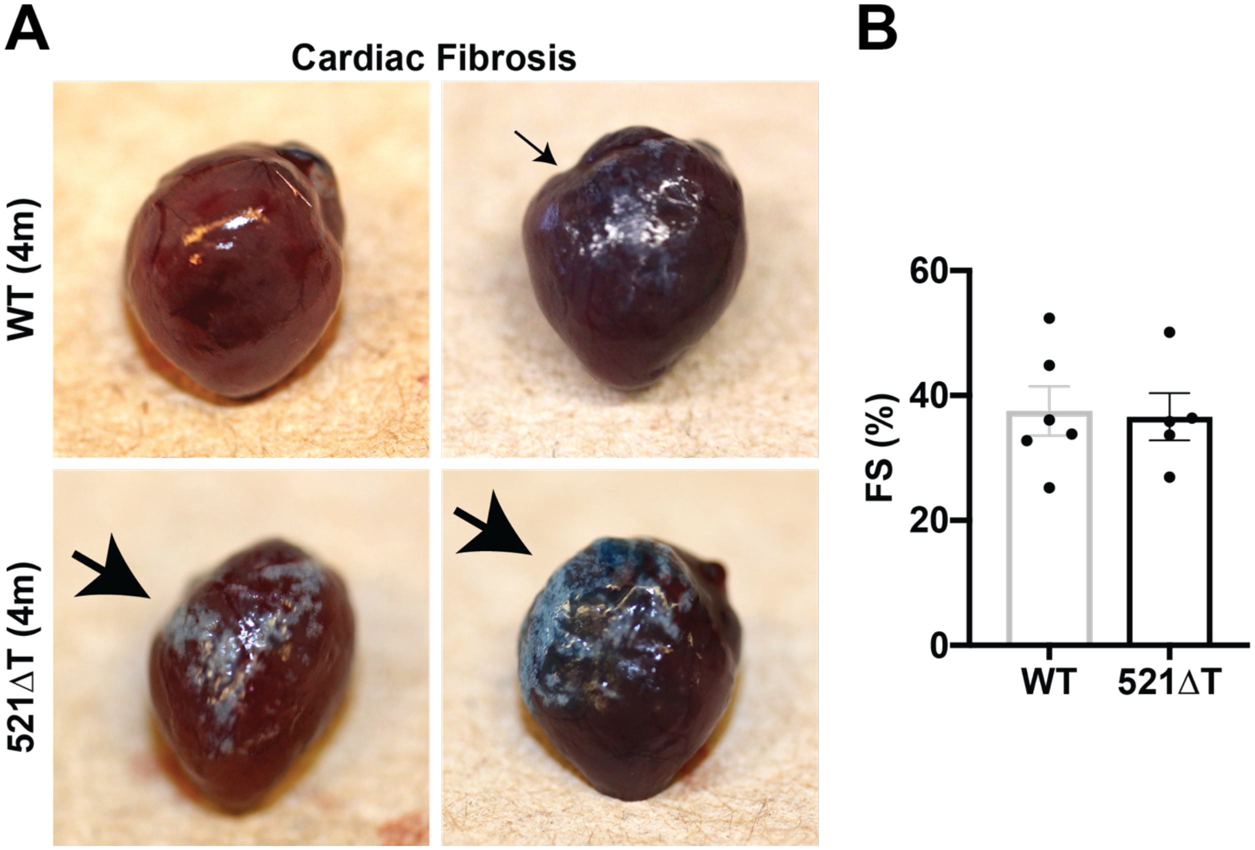

Figure S4. Enhanced ventricular fibrosis in 521ΔT hearts. Gross imaging reveals enhanced ventricular fibrosis (white patches, marked by black arrow) in 521ΔT hearts at 4-months of age. Of note, wildtype hearts on the DBA/2J background present with small amounts of visible fibrotic lesions at 4-months of age (top panel, small black arrow). Despite these fibrotic patches, percent fractional shortening was not significantly different between genotypes at 4-months of age. n=5, t-test.

Disease Models & Mechanisms • Supplementary information
